# Three Ambient Listening Systems in Simulated Dutch-Language Multispeaker and Multidialect Clinical Encounters: Comparative Study

**DOI:** 10.2196/94586

**Published:** 2026-10-16

**Authors:** Anne H. Hoekman, Tarannom Mehri, Job N. Doornberg, Mirjam Plantinga, Tom P. van der Laan, Charlotte M.H.H.T. Bootsma-Robroeks, Rosanne C. Schoonbeek

**Affiliations:** 1 Department of Health Information Office, Information Management Healthcare University Medical Center Groningen Groningen The Netherlands; 2 Department of Trauma Surgery / Orthopedics University Medical Center Groningen Groningen The Netherlands; 3 Department of Orthopaedic Trauma Flinders University Adelaide Australia; 4 ELSA AI Lab Northern Netherlands, University Medical Center Groningen University of Groningen Groningen The Netherlands; 5 Department of Genetics and Data Science Center in Health, University Medical Center Groningen University of Groningen Groningen The Netherlands; 6 Department of Otolaryngology – Head and Neck Surgery University Medical Center Groningen Groningen The Netherlands; 7 Department of Pediatrics, Pediatrics Nephrology, Beatrix Children’s Hospital University Medical Center Groningen Groningen The Netherlands

**Keywords:** large language models, generative artificial intelligence, generative AI, ambient listening, electronic health records, artificial intelligence adoption, AI adoption

## Abstract

**Background:**

Clinicians spend a lot of time on electronic health record (EHR) documentation, often at the expense of direct patient interaction. Ambient listening technology uses AI to passively record and summarize clinical encounters. Although early studies are promising, there is limited evidence on system performance in complex, non-English settings.

**Objective:**

This study aimed to compare the documentation performance of 3 commercially available ambient listening systems in simulated Dutch-language outpatient consultations by assessing note completeness, correctness, and conciseness under predefined linguistic and interactional challenges.

**Methods:**

Standardized audio recordings of 10 scripted physician-patient interactions across 2 specialties were used. Scenarios included multispeaker dynamics (patient companion), conversational disruptions (nurse interruption), evasive patient communication, and a regional dialect (Gronings). Three ambient listening providers (provider 1, provider 2, and provider 3) processed the audio files. Generated notes were divided into 4 note sections: patient history, physical examination, diagnostic impression, and treatment plan. For each system output, each note section was scored for completeness, conciseness, and correctness using a 5-point ordinal scale against physician-developed reference summaries. In total, 8 clinicians participated in scoring: 4 junior clinicians evaluated all outputs, and 4 senior clinicians each reviewed a subset of scenarios. Analyses were based on 351 rater-averaged section-domain scores. Interrater agreement was assessed using Gwet agreement coefficient 2 (Gwet AC-2). System-level technical characteristics were assessed alongside clinical performance to aid interpretation of between-provider differences.

**Results:**

Across 351 rater-averaged section-domain scores on a 5-point scale, overall interrater agreement was high (Gwet AC-2=0.827). Provider-level mean scores ranged from 3.82 to 4.26 (4.26, SD 0.73 for provider 3, 4.00, SD 0.90 for provider 2, and 3.82, SD 1.07 for provider 1). Mean scores were higher in otolaryngology than in surgical oncology (4.36, SD 0.56 vs 3.68, SD 1.10). Across scoring domains, correctness received the highest mean score (4.21, SD 0.98), while completeness received the lowest (3.81, SD 0.90). Variation in mean scores was observed across script scenarios. Dialect-containing scenarios had the lowest mean score overall (3.77, SD 1.21), although this pattern was not uniform across specialties and systems. Median summary generation times ranged from 13.5 seconds (IQR 12-15; provider 3) to 22.0 seconds (IQR 18-24; provider 2).

**Conclusions:**

Ambient listening systems demonstrated generally strong performance in this simulated Dutch-language evaluation, including under several common conversational challenges. However, mean scores varied across scripted scenarios, with lower scores observed in some dialect-containing and otherwise challenging interactions. Future deployment studies must prioritize linguistic equity, real-world validation of efficiency gains, and evaluation of both clinician and patient perceptions to understand how these systems influence consultation dynamics and care delivery across diverse patient populations.

## Introduction

Clinical documentation during patient encounters remains a significant challenge. Physicians often face a trade-off between documenting in real time, which may undermine face-to-face interaction, and postponing documentation, which leads to working in “pajama time” and delayed record completion. Studies have shown that physicians may spend nearly half of their workday interacting with the electronic health record (EHR), sometimes leaving only 27% for direct patient care. This administrative burden is strongly associated with reduced job satisfaction and increased burnout [1-6].

The consequences extend beyond clinicians. When attention is directed toward a computer screen rather than the patient, consultations may feel less personal [7]. Reduced eye contact and increased cognitive load during documentation have been shown to affect trust, communication quality, and patient satisfaction [8,9].

Ambient listening technology has emerged as a potential solution. These systems passively capture physician-patient conversations and use AI to transcribe and summarize encounters directly into structured clinical notes. Early evaluations suggest reductions in documentation burden, after-hours charting, and clerical workload [10-17]. However, evidence for patient-related benefits remains limited and heterogeneous [18]. Importantly, most existing evaluations have been conducted in English-language settings [19].

Less is known about how these systems perform in other languages when consultations include multiple speakers, conversational interruptions, indirect or evasive patient communication, and regional dialect use.

Our study evaluates the performance of 3 ambient listening systems in scripted Dutch-language consultations before clinical deployment. Because ambient listening systems differ substantially in architecture and integration, we additionally examined key technical characteristics to support the interpretation of performance differences and real-world adoption decisions. The scenarios incorporated interactional challenges, including multispeaker dialogue, conversational disruption, evasive communication, and the use of a regional dialect (Gronings), to assess the linguistic and contextual robustness of these systems. By exploring these dimensions, our study contributes novel insights into the linguistic and contextual adaptability of ambient AI documentation tools and their potential applicability in diverse real-world health care settings.

## Methods

### Scripted Audio Design

The study used audio recordings of scripted physician-patient interactions in 2 clinical domains: otolaryngology and surgical oncology. These domains were selected to include both relatively routine outpatient consultations and more complex oncological conversations at a high-volume oncological center, while keeping the pilot evaluation feasible. All scripts were written by physicians but were fully fictional, incorporating invented names, clinical cases, and dialogues. A total of 10 distinct scripts were developed, each involving up to 4 roles: physician, patient, patient companion, and nurse. Departmental staff volunteered to perform these roles and provided informed consent for the recording, storage, and use of their audio data. A total of 4 unique speakers participated in the recordings, including 2 clinicians and 2 nonclinicians. Within a given script, each speaker performed only 1 role, and there was a maximum of 4 roles per script. All speakers were fluent in Dutch. For the dialect-specific scenario, the participant voicing the Gronings-speaking patient was familiar with this dialect through their regional background. We aimed to reflect a realistic mix of speaker roles and consultation dynamics that may occur in outpatient practice. The scripts were shared with participating staff in advance to allow them to become familiar with the content. Immediately prior to recording, participants reviewed the scripts again. During recording, the interactions were primarily script-based rather than fully improvised; however, participants were encouraged to deliver the dialogue naturally, and small variations, such as filler words, minor phrasing adjustments, and limited natural overlap where consistent with the script, were allowed where appropriate. No substantial deviations from the scripted content were made.

### Script Design and Structure

Two sets of 5 scripts were created: set 1 for otolaryngology and set 2 for surgical oncology, numbered 1.1 to 1.5 and 2.1 to 2.5, respectively (Multimedia Appendices 1 and 2). The scripts were constructed to reflect different consultation dynamics that may occur during routine clinical encounters. These interactional challenges were selected to introduce variation and to represent situations encountered in outpatient practice that may affect ambient listening performance. The aim was to evaluate system performance not only in straightforward consultations but also in conversational circumstances that may be more challenging for transcription and summarization. Each script type represented a distinct interactional scenario:

- Baseline scripts (1.1 and 2.1): standard Dutch consultations, including patient history, physical examination, diagnostic impression, and treatment plan, without interruptions or additional speakers
- Companion-influenced scripts (1.2 and 2.2): consultations in which a patient companion actively participated in the dialogue
- Disrupted scripts (1.3 and 2.3): consultations interrupted by a nurse entering the room, resulting in conversational disruption and early termination
- Evasive patient scripts (1.4 and 2.4): consultations in which the patient frequently digressed or provided irrelevant information
- Dialect-specific scripts (1.5 and 2.5): consultations conducted in Gronings, a regional Dutch dialect. This scenario was included because regional language variation is relevant in our clinical setting in the north of the Netherlands. It was designed to explore whether such variation influenced ambient listening performance and to ensure that the evaluation reflected speech variation that may be encountered in routine care at our institution rather than standardized Dutch alone. No predefined list of anticipated problematic words or phrases was specified in advance.

English translations of the baseline otolaryngology and surgical oncology scripts are provided in Multimedia Appendix 3.

### Ambient Listening Providers

Ambient listening providers were recruited via an open call to companies collaborating with our hospital. All interested providers were invited to participate. Three providers participated in the evaluation: Autoscriber (Autoscriber BV; provider 1), Juvoly powered by Tandem (Tandem Health; provider 2), and OurMind (OurMind BV; provider 3). In this manuscript, “provider” refers to the company providing the ambient listening tool, whereas “clinician” or “physician” refers to the health care professional evaluating the outputs. Throughout the remainder of the manuscript, the providers are referred to as provider 1, provider 2, and provider 3 to maintain consistency across the Results section, Tables, and Figure. Providers were informed about the general testing process but were not given access to the script content. They received a high-level outline of the expected summary structure: patient history, physical examination, diagnostic impression, and treatment plan.

### Recording and Playback Protocol

Recordings were conducted at the University Medical Center Groningen in a private, soundproof room using a Windows-based laptop. This was a soundproof meeting room, rather than a studio environment. During recordings, the microphone was positioned centrally between the physician and the patient-side speakers. Audio recordings were stored on a secure institutional research drive.

Playback conditions were identical for all providers: recordings were played from the same Microsoft PC (Microsoft Corp) connected to a Logitech external speaker (Logitech International SA). At the start of each session, the provider’s ambient listening system was activated through its standard interface, and generated summaries were saved for evaluation. During testing, audio files were played locally on the research computer while the ambient listening providers captured the sound through their standard audio input. The providers did not store any audio data.

### Reference Standard

For benchmarking, 2 physician-authors (AHH and RCS) developed reference summaries a priori for each specialty. Reference summaries were initially drafted separately and then iteratively merged through review of commented versions until a final agreed version was reached. Within each specialty, the baseline, companion-influenced, evasive, and dialect scenarios shared the same underlying consultation content and therefore used the same reference summary structure. The disrupted scenario was intentionally shortened and therefore required a separate reference summary. The reference summaries defined the key expected elements for each scenario, namely the core clinically relevant content expected in the note according to the common outpatient note format used in our hospital: patient history, physical examination, diagnostic impression, and treatment plan. In the disrupted scenarios, elements that were not reached during the consultation were intentionally not included in the reference summary. The reference summaries were used to standardize rater comparisons across providers. The reference summaries were not shared with the providers.

### Evaluation Framework

Each generated summary was divided into 4 clinical note sections: patient history (including presenting complaint), physical examination, diagnostic impression, and treatment plan. These sections were chosen because they represent common and clinically meaningful components of outpatient documentation in both otolaryngology and surgical oncology and broadly reflect how notes are structured in the EHR in these specialties at our institution. Each section was evaluated on 3 domains: completeness, correctness, and conciseness, using a 5-point ordinal scale and compared with the reference summary [20]. On the 5-point ordinal scale, a score of 1 indicated no usable output or severely inadequate content, while a score of 5 indicated near-complete, accurate, and concise documentation.

Four junior clinicians evaluated all outputs. Each scenario was additionally reviewed by 1 senior clinician with at least 5 years of clinical experience, ensuring senior clinical oversight of the specialty-specific content.

For analysis, the unit of observation was the section-domain score aggregated across raters. For each provider output, ratings from the available reviewers were averaged for each note section and evaluation domain. In 1 disrupted script (2.3), the physical examination was intentionally omitted and therefore not scored. Consequently, rather than the theoretical 360 section-domain evaluations (10 scenarios×3 providers×4 sections×3 domains), a total of 351 aggregated scores were obtained. These aggregated section-domain scores formed the basis for all subsequent analyses.

### Technical Characteristics

In addition to documentation quality, each provider’s technical characteristics were assessed, including processing speed, system requirements, integration capability, data security and privacy compliance, model architecture, and template customization options.

### Statistical Analysis

Descriptive statistics were used to summarize case characteristics, clinician rating scores, and provider processing times. Because summary quality scores were recorded on a 5-point Likert scale, they were treated as ordinal. Interrater reliability was quantified using Gwet agreement coefficient 2 (Gwet AC-2) with quadratic weights rather than intraclass correlation coefficients, and agreement estimates are reported with 95% CIs.

For descriptive comparison across providers, specialties, domains, and note sections, rating scores are presented as means with SDs and medians with IQRs. Processing times were summarized using means with SDs and medians with IQRs; medians were emphasized due to right-skewed distributions and the presence of outliers. No inferential statistical comparisons between providers were performed, given the comparative and predeployment nature of this evaluation.

All domains and note sections were weighted equally. Analyses were conducted using R (version 4.5.1; R Foundation for Statistical Computing) and Stata (version 18.0; StataCorp LLC).

### Ethical Considerations

This study was conducted under the EPICAI (Implementation of generative artificial intelligence in Epic—a scientific analysis of risks and benefits) research protocol (UMCG RR 19035; METc 2024/098), which was reviewed by the Medical Ethics Review Board of the University Medical Center Groningen. The board determined that the protocol did not constitute research with human subjects as defined by the Dutch Medical Research Involving Human Subjects Act (WMO) and therefore did not require full ethical review or approval. All clinical encounters were simulated using entirely fictional patient scenarios; no actual patients, patient data, or EHR data were involved. Colleagues participated as actors and provided informed consent for audio recording and subsequent processing of the recordings for research purposes. Recordings were stored on the institution's secure research drive with access restricted to authorized research personnel. Participants received no additional compensation for participation; participation occurred during working hours.

## Results

### Provider-, Section-, and Domain-Specific Scores

Each provider-generated note contained structured note sections (patient history, physical examination, diagnostic impression, and treatment plan) derived from the recorded consultation. Each section was independently scored on 3 domains (completeness, conciseness, and correctness). Across 351 rater-averaged section-domain scores, provider-level means were 4.26, SD 0.73 for provider 3, 4.00, SD 0.90 for provider 2, and 3.82, SD 1.07 for provider 1 (Table 1). Averaged across scenarios, mean scores were higher in otolaryngology than in surgical oncology (4.36, SD 0.56 vs 3.68, SD 1.10). Section-specific means showed limited overall spread: 4.13, SD 0.72 for treatment plan, 4.11, SD 1.12 for diagnostic impression, 3.97, SD 1.09 for physical examination, and 3.90, SD 0.69 for patient history. Across domains, correctness scored highest, followed by conciseness, and completeness scored lowest (4.21, SD 0.98, 4.07, SD 0.86, and 3.81, SD 0.90, respectively). Averaged across specialties, scenario means ranged from 3.77, SD 1.21 for script 5 to 4.18, SD 0.73 for script 4, with intermediate values for scripts 1 to 3 (3.98, SD 0.91, 4.09, SD 0.88, and 4.14, SD 0.77 respectively). As shown in Figure 1, this pattern was consistent across providers, with lower mean scores observed for dialect-specific scenarios than other scenario types. Scenario 5 (dialect) showed the lowest mean and the highest variability.

Table 2 illustrates how differences in provider performance translated into clinically relevant variation in the generated treatment plans. Provider 1 achieved the highest mean score by covering the key diagnostic, surgical, and care coordination elements specified in the a priori reference plan, including staging imaging, operative planning, and oncology nursing follow-up. Provider 3 documented appropriate staging and operative planning but did not mention complication counseling and oncology nurse assignment, contributing to a modestly lower score. Provider 2 documented surgical planning but did not capture essential staging imaging elements, and reviewers rated the plan lower.

Detailed section-level scores for each provider across all script scenarios and both specialties are provided in Multimedia Appendices 1 and 2.

**Table 1:** Descriptive summary of rater-averaged section-domain scores by provider, specialty, note section, domain, and script^a^.

Categories and subgroups^b^			Observations, n		Score, mean (SD)		Score, median (IQR)
**Provider**							
	Provider 1	117		3.82 (1.07)		4.20 (3.40-4.60)	
	Provider 2	117		4.00 (0.90)		4.20 (3.60-4.60)	
	Provider 3	117		4.26 (0.73)		4.40 (4.00-4.80)	
**Specialty**							
	Otolaryngology	180		4.36 (0.56)		4.40 (4.20-4.80)	
	Surgical oncology	171		3.68 (1.10)		4.00 (3.20-4.60)	
**Note section**							
	Patient history	90		3.90 (0.69)		4.00 (3.40-4.40)	
	Physical examination	81		3.97 (1.09)		4.40 (3.80-4.60)	
	Diagnostic impression	90		4.11 (1.12)		4.40 (4.00-4.80)	
	Treatment plan	90		4.13 (0.72)		4.30 (3.80-4.80)	
**Domain**							
	Completeness	117		3.81 (0.90)		4.00 (3.40-4.40)	
	Correctness	117		4.21 (0.98)		4.60 (4.00-4.80)	
	Conciseness	117		4.07 (0.86)		4.20 (3.80-4.60)	
**Script number**							
	1	72		3.98 (0.91)		4.20 (3.60-4.60)	
	2	72		4.09 (0.88)		4.40 (3.90-4.60)	
	3	63		4.14 (0.77)		4.20 (3.80-4.80)	
	4	72		4.18 (0.73)		4.40 (3.80-4.80)	
	5	72		3.77 (1.21)		4.20 (3.20-4.60)	

^a^Scores were rated on a 5-point ordinal scale, with higher scores indicating better performance. Values are based on rater-averaged section-domain scores and are presented as mean (SD) and median (IQR). n indicates the number of rater-averaged section-domain scores.

^b^The physical examination section was omitted in surgical oncology, script 2.3; therefore, fewer ratings contributed to the surgical oncology specialty summary, physical examination note section summary, and script 3 summary.

**Figure 1:**
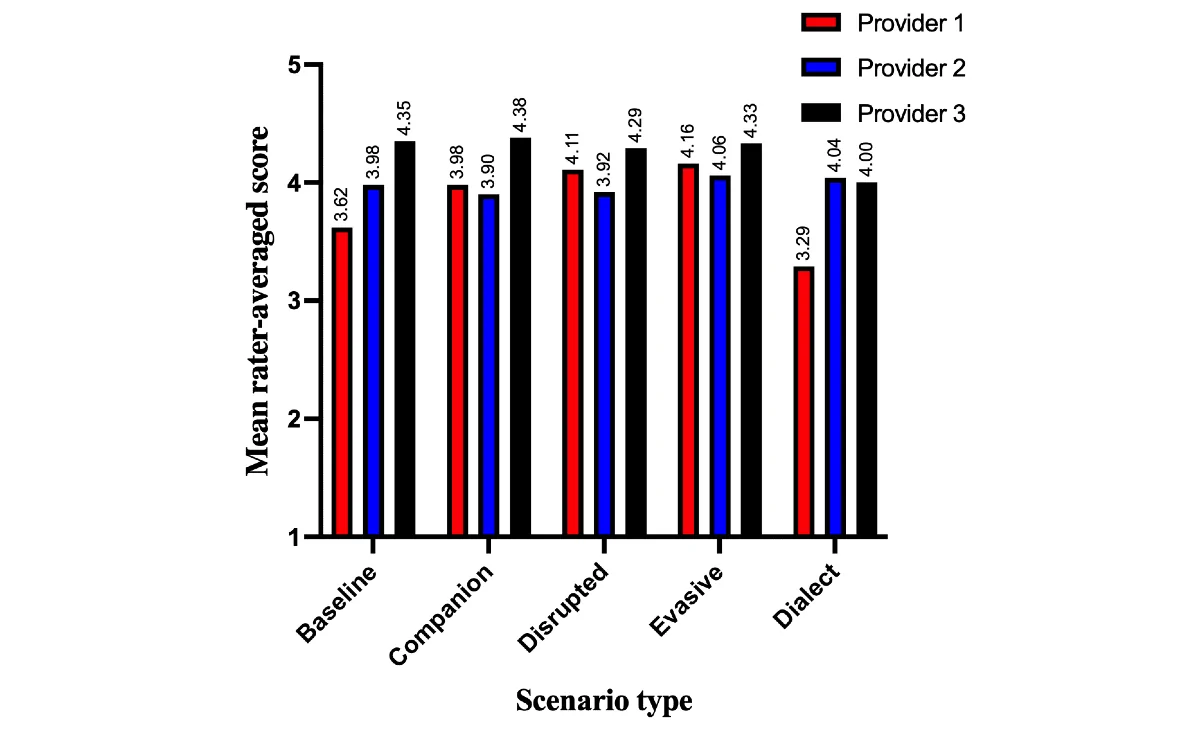
Provider performance across scenario types. Bars represent mean rater-averaged section-domain scores on a 5-point scale for each provider across scenario types. For each scenario type, scores were averaged across the corresponding otolaryngology and surgical oncology scripts and across evaluated note sections and domains. Specialty-specific results are shown in Multimedia Appendices 1 and 2.

**Table 2:** Example comparison of treatment plans generated by 3 ambient listening systems for script 2.4 (surgical oncology; evasive patient), presented as an English translation. Scores are based on rater-averaged evaluations of completeness, correctness, and conciseness relative to the clinician-developed reference plan and are reported on a 5-point scale^a^.

Providers	Treatment plan (English translation)	Rater-averaged treatment plan score
Provider 1	CTb scan of the abdomen and chest MRIc of the pelvis for preoperative screening Planned sigmoid resection Consideration of chemotherapy depending on disease stage and findings Smoking cessation if applicable Healthy diet Moderate-intensity physical activity Contact with oncology nurse specialist	4.4
Provider 2	Colectomy with primary anastomosis within 2 weeks Possible chemotherapy depending on disease stage and lymph node status Smoking cessation Healthy diet Moderate-intensity physical activity Preoperative screening	3.0
Provider 3	CT scan of the abdomen and chest to assess for metastases MRI of the pelvis to evaluate local tumor extension Planned sigmoid resection with primary anastomosis Preoperative screening to assess surgical fitness Consideration of chemotherapy based on imaging and surgical findings	4.1

^a^All systems were evaluated using the same recorded audio file. For this illustrative treatment plan section, ratings from the available reviewers were averaged within each evaluation domain; the displayed score summarizes the resulting rater-averaged section-domain scores across completeness, correctness, and conciseness. The a priori reference management plan specified the following expected elements: scheduling CT of the chest or abdomen and MRI of the pelvis, discussion of results at a multidisciplinary tumor board, scheduling sigmoid resection with complications discussed, consideration of chemotherapy depending on imaging and operative findings, and assignment of an oncology nurse specialist.

^b^CT: computed tomography.

^c^MRI: magnetic resonance imaging.

### Provider Performance Across Scenario Types

Bars represent mean rater-averaged section-domain scores on a 5-point scale for each provider across scenario types. For each scenario type, scores were averaged across the corresponding otolaryngology and surgical oncology scripts and across evaluated note sections and domains. Specialty-specific results are shown in Multimedia Appendices 1 and 2.

To complement the quantitative ratings and the illustrative example in Table 2, we descriptively reviewed recurrent qualitative error patterns across system outputs. These included omissions of clinically relevant information, loss of specificity, hallucinations, misinterpretations, unnecessary information, redundancy, and overelaboration. Representative examples are summarized in Table 3.

**Table 3:** Recurrent qualitative error categories observed across summaries, with illustrative examples.

Domains and error categories			Examples
**Completeness**			
	Omission of timing	“Follow-up” instead of “Follow-up in 6 weeks”	
	Omission of key clinical content	Missing relevant negatives, medication, physical examination, diagnostic impression, oncology nurse involvement, or multidisciplinary oncology discussion	
	Omission of conditionality	Chemotherapy without “depending on scan and operative findings”	
	Omission of laterality	Dix-Hallpike without “right-sided”	
**Correctness**			
	Hallucination	Not observed	
	Loss of medical terminology	“Had the tonsils taken out” instead of “tonsillectomy”	
	Misinterpretation	“Adenocarcinoma in the caecum” instead of sigmoid	
**Conciseness**			
	Unnecessary information	Irrelevant remarks about football club membership or global problems	
	Redundancy	Repeated information across headings, for example, smoking, alcohol, and drug use in both social history and intoxications	
	Overelaboration	Detailed description of benign paroxysmal positional dizziness pathophysiology	

### Interrater Agreement

Overall agreement (Gwet AC-2 score) across 8 raters was 0.83 (95% CI 0.81-0.85), indicating good to very good concordance. Agreement among juniors alone was similar (Gwet AC-2=0.83, 95% CI 0.81-0.85). For combinations of the junior raters with a single senior rater, Gwet AC-2 ranged from 0.80 to 0.85 across seniors. By domain, agreement was highest for correctness (Gwet AC-2=0.86, 95% CI 0.82-0.90), followed by conciseness (0.85, 95% CI 0.81-0.88) and completeness (0.78, 95% CI 0.74-0.81). By section, agreement was highest for diagnostic impression (0.88, 95% CI 0.85-0.92), followed by physical examination (0.84, 95% CI 0.80-0.87), treatment plan (0.82, 95% CI 0.77-0.87), and patient history (0.78, 95% CI 0.73-0.83). Detailed interrater agreement estimates stratified by rater group, scoring domain, and note section are provided in Multimedia Appendix 4.

### Generation Time (Seconds)

Across scripts, summaries were typically produced within 10 to 36 seconds, with a small number of outliers. Mean generation times by provider were 15.6, SD 7.4 seconds for provider 3, 26.9, SD 18.0 seconds for provider 2, and 63.3, SD 106.4 seconds for provider 1 (overall mean 35.3, SD 63.7 seconds). Two provider 1 scripts (1.4 and 2.4) and 1 provider 2 script (2.5) exceeded 1 minute (Table 4). Median generation times were 13.5 seconds (IQR 12-15) for provider 3, 22.0 seconds (IQR 18-24) for provider 2, and 17.5 seconds (IQR 12-30) for provider 1, with an overall median of 17.5 seconds (IQR 13-23).

**Table 4:** Processing time for AI-generated summaries across scenarios and providers^a^.

Scripts	Provider 1, processing time (seconds)	Provider 2, processing time (seconds)	Provider 3, processing time (seconds)
1.1 Otolaryngology—baseline	6	29	36
1.2 Otolaryngology—companion	12	18	14
1.3 Otolaryngology—disrupted	12	24	16
1.4 Otolaryngology—evasive	332	19	15
1.5 Otolaryngology—dialect	20	23	15
2.1 Surgical oncology—baseline	15	18	12
2.2 Surgical oncology—companion	30	17	12
2.3 Surgical oncology—disrupted	23	23	10
2.4 Surgical oncology—evasive	171	21	13
2.5 Surgical oncology—dialect	12	77	13

^a^Processing time was defined as the interval from initiating summary generation (“generate summary”) to receipt of the finalized AI-generated summary.

### Overview of Provider Performance and Technical Characteristics

Beyond processing speed and clinical performance, the evaluated transcription software systems exhibited differences in their underlying architecture. All 3 systems had minimal and identical system requirements, needing only a browser, microphone, and internet connection for operation. However, meaningful distinctions were observed in processing speed, model training background, and template customization options. Provider 3 demonstrated the fastest processing time, whereas provider 2 showed slower generation times. Differences were also observed in the reported language model architecture. Provider 2 reported a Dutch-language speech-to-text model, whereas provider 3 used an English-language model with Dutch compatibility within a multilayer pipeline. Provider 1 also used a Dutch-language speech-to-text engine, and no translation step was reported. Template customization was possible in all systems, although the reported flexibility differed at the provider, institutional, departmental, and clinician levels. Reported security and compliance characteristics were broadly similar in that all systems were described as General Data Protection Regulation (GDPR)–compliant and Nederlandse Norm (NEN; Dutch Standard) 7510/ISO 27001–aligned, but additional certifications varied among providers. In the present study, no audio data were stored by the providers. These evaluation results and provider-reported technical characteristics are summarized in Table 5.

**Table 5:** Evaluation results and provider-reported specifications of the 3 ambient listening providers^a^.

Features			Provider 1		Provider 2		Provider 3
**Part 1: evaluation results (December 2024)**							
	Overall rater-averaged section-domain score (5-point), mean (SD)	3.82 (1.07)		4.00 (0.90)		4.26 (0.73)	
	Processing time (seconds), median (IQR)	17.5 (12-30)		22.0 (18-24)		13.5 (12-15)	
**Part 2: provider-reported specifications (May 2026)**							
	Integration with Epic	Yes		Yes		Yes	
	System requirements	Browser, microphone, and internet		Browser, microphone, and internet		Browser, microphone, and internet	
	Security, compliance, and certification	ISO 27001, NEN^b^ 7510, GDPR^c^-compliant, and QMS^d^ based on ISO 13485		ISO 27001, ISO 13485, ISO 42001, ISO 14001, NEN 7510, GDPR-compliant, and MDR^e^ class IIa certified		ISO 27001, NEN 7510, and GDPR-compliant	
	Language model	Dutch-language speech-to-text engine (no translation step reported)		Dutch-language speech-to-text model		English-language model with Dutch compatibility, used within a multilayer pipeline	
	Template support and customization	Fully customizable; template builder available; custom templates available at institutional, departmental, and clinician levels		Customizable up to the clinician level, with support before, during, and after implementation; template builder and template library available		Customizable at provider, institutional, and clinician level; community template library planned	

^a^Evaluation results reflect the provider versions tested in December 2024. Provider-reported specifications reflect information verified by the providers in May 2026 and may not fully correspond to the configurations evaluated in December 2024. Overall mean scores are based on rater-averaged section-domain observations across all scenarios, note sections, and evaluation domains, with higher scores indicating better performance. Processing time is reported as the median time from initiating summary generation to receipt of the finalized AI-generated summary.

^b^NEN: Nederlandse Norm (Dutch Standard).

^c^GDPR: General Data Protection Regulation.

^d^QMS: quality management system.

^e^MDR: Medical Device Regulation.

## Discussion

### Principal Findings

Ambient listening systems are increasingly proposed to reduce documentation burden and support clinicians in high-volume, digitally intensive care environments [21,22]. Although early evaluations suggest potential efficiency and workflow benefits, evidence remains limited regarding system performance outside English-speaking settings and during consultations involving complex interactional dynamics [14].

This study evaluated 3 ambient documentation systems in Dutch-language consultations that incorporated multiple speakers, conversational disruption, evasive dialogue, and regional dialect use. All systems were able to generate clinically usable draft summaries across most scenarios, demonstrating that ambient listening technology can function effectively beyond English-language environments. Variation in mean scores was observed across scenarios and specialties, with dialect scenarios generally associated with lower scores and greater variability.

Importantly, these outputs are intended to support clinicians and require human review. Although overall mean scores were high, the observed variability indicates that some summaries were substantially weaker, which may translate into a greater editing burden in practice and highlights the need for appropriate implementation safeguards. In practice, this implies multiple behavioral adjustments for clinicians: careful review of generated documentation and more explicit verbalization of examination findings and clinical reasoning during the encounter, as ambient listening systems rely entirely on spoken dialogue to produce their summaries.

Direct comparison with prior studies is limited because evaluation instruments and scoring methods differ widely across the ambient documentation literature. A recent scoping review highlighted the lack of standardized metrics used to evaluate ambient clinical documentation systems and emphasized the need for more structured evaluation approaches [19]. In this context, our findings align with broader evidence showing that ambient systems can produce usable draft documentation while remaining sensitive to linguistic variation and conversational complexity.

Dialect-containing scenarios tended to receive lower ratings in our dataset, although this pattern was not uniform across specialties and providers. The lower scores observed in these scenarios may indicate that regional language variation can influence ambient listening performance in our setting, but this finding should be interpreted cautiously. Differences may also have been shaped by specialty-specific script content or other contextual features of the scenarios.

Taken together, these findings provide insight into how ambient listening systems behave under varied consultation dynamics in a predeployment setting.

### Strengths

A key strength of this study is the direct, side-by-side comparison of multiple commercial ambient documentation systems using identical audio input and standardized evaluation criteria. Evaluation across 2 specialties and varied consultation dynamics further strengthens the relevance of the findings. High interrater agreement supports the robustness of the assessment framework.

### Limitations

This study used scripted consultations, which cannot fully replicate the unpredictability of live clinical encounters. Although the recordings were performed by clinicians and delivered naturally where possible, they remained primarily script-based and may not have fully captured features of spontaneous speech. Playback also took place under controlled acoustic conditions in a soundproof meeting room. Although this supported standardized side-by-side comparison across systems, and the scripted scenarios incorporated realistic interactional challenges such as interruptions and multispeaker dialogue, it does not fully capture the acoustic complexity of routine clinical care, including background noise, room acoustics, microphone positioning, and unstructured workflow interruptions. The simulated design also involved a limited number of speakers and therefore may not reflect the full range of speaker variation encountered in routine clinical practice. Evaluation of documentation quality also involves subjective judgment, despite strong interrater reliability. In addition, only 1 regional dialect was evaluated, and multilingual consultations were not included. Finally, as a simulated predeployment evaluation, this study does not assess actual workflow impact or clinician time savings.

### Future Directions

Future research should focus on real-world deployment studies in routine clinical practice, where both objective outcomes (such as actual documentation time and editing burden) and subjective outcomes (such as clinician experience and patient perceptions) can be assessed. Expanding evaluations to multilingual consultations and a broader range of dialects is essential to ensure equitable performance across diverse patient populations. In parallel, ethical and legal considerations, including transparency, patient consent, and responsibility for documentation errors, must be addressed as these systems move toward wider clinical adoption.

### Conclusions

In summary, this study shows that ambient listening systems can achieve strong performance in non-English clinical settings, even under conversationally complex conditions. Although linguistic variation remains a key challenge, the ability of multiple systems to produce clinically acceptable documentation suggests the potential for broader international adoption. Continued evaluation in real-world, linguistically diverse environments will be essential for safe and effective implementation.

## Appendix

Multimedia Appendix 1 Section-level documentation quality scores by script scenario and provider in otolaryngology.

Multimedia Appendix 2 Section-level documentation quality scores by script scenario and provider in surgical oncology.

Multimedia Appendix 3 Baseline scripts for otolaryngology and surgical oncology (English translation).

Multimedia Appendix 4 Interrater agreement for clinical documentation quality scores using Gwet agreement coefficient 2 (Gwet AC-2).

## Abbreviations

EHR: electronic health record
EPICAI: Epidemiology and Care in Acne inversa
GDPR: General Data Protection Regulation
Gwet AC-2: Gwet agreement coefficient 2
NEN: Nederlandse Norm
WMO: Medical Research Involving Human Subjects Act

## Data Availability

The datasets generated and analyzed during this study are not publicly available due to contractual restrictions with commercial system providers and institutional data governance policies. Deidentified rating data and aggregated performance metrics may be made available by the corresponding author upon reasonable request and subject to institutional approval.
