# Supplementary material for: Three Ambient Listening Systems in Simulated Dutch-Language Multispeaker and Multidialect Clinical Encounters: Comparative Study
Source: JMIR Med Inform. 2026 Oct 16;14:e94586. doi: 10.2196/94586 (PMC13722924; doi:10.2196/94586)
Supplement: Supplementary file 1 [file medinform_v14i1e94586_app1.docx]

**Multimedia Appendix 1. Section-level documentation quality scores by script scenario and provider in otolaryngology.**

| Script scenario | Provider | Patient history | Physical examination | Diagnostic impression | Treatment plan | Overall mean across note sections |
| --- | --- | --- | --- | --- | --- | --- |
| 1.1 – Baseline | |  |  |  |  |  |
|  | Provider 1 | 4.47 | 4.27 | 3.73 | 3.40 | 3.97 |
|  | Provider 2 | 4.33 | 3.60 | 4.47 | 4.13 | 4.13 |
|  | Provider 3 | 3.60 | 4.53 | 4.73 | 4.27 | 4.28 |
| 1.2 – Companion | |  |  |  |  |  |
|  | Provider 1 | 4.47 | 4.13 | 4.47 | 4.47 | 4.38 |
|  | Provider 2 | 4.40 | 3.93 | 4.53 | 4.73 | 4.40 |
|  | Provider 3 | 4.47 | 4.60 | 4.53 | 4.47 | 4.52 |
| 1.3 – Disrupted | |  |  |  |  |  |
|  | Provider 1 | 4.00 | 3.87 | 3.73 | 4.80 | 4.10 |
|  | Provider 2 | 4.20 | 4.73 | 4.80 | 4.73 | 4.62 |
|  | Provider 3 | 3.73 | 4.67 | 4.73 | 4.07 | 4.30 |
| 1.4 – Evasive | |  |  |  |  |  |
|  | Provider 1 | 4.27 | 4.33 | 4.27 | 4.87 | 4.43 |
|  | Provider 2 | 3.93 | 4.47 | 4.67 | 4.33 | 4.35 |
|  | Provider 3 | 4.47 | 3.73 | 4.67 | 4.33 | 4.30 |
| 1.5 – Dialect (Gronings) | |  |  |  |  |  |
|  | Provider 1 | 3.93 | 4.07 | 4.67 | 4.40 | 4.27 |
|  | Provider 2 | 4.60 | 4.40 | 4.73 | 4.07 | 4.45 |
|  | Provider 3 | 4.67 | 4.93 | 4.93 | 4.93 | 4.87 |

Values represent mean rater-averaged scores on a 1–5 scale, with higher scores indicating better performance. For each provider and script scenario, note-section scores summarize rater-averaged section–domain evaluations across completeness, correctness, and conciseness. The overall mean represents the mean across the evaluated note sections. A score of 1 indicates that no usable output was generated for the corresponding note section and was therefore assigned the lowest possible value.
