# Supplementary material for: Three Ambient Listening Systems in Simulated Dutch-Language Multispeaker and Multidialect Clinical Encounters: Comparative Study
Source: JMIR Med Inform. 2026 Oct 16;14:e94586. doi: 10.2196/94586 (PMC13722924; doi:10.2196/94586)
Supplement: Supplementary file 2 [file medinform_v14i1e94586_app2.docx]

| Script scenario | Provider | Patient history | Physical examination | Diagnostic impression | Treatment plan | Overall mean across note sections |
| --- | --- | --- | --- | --- | --- | --- |
| 2.1 – Baseline | |  |  |  |  |  |
|  | Provider 1 | 3.60 | 4.13 | 1.00 | 4.33 | 3.27 |
|  | Provider 2 | 3.53 | 4.33 | 4.60 | 2.87 | 3.83 |
|  | Provider 3 | 4.33 | 4.27 | 4.87 | 4.20 | 4.42 |
| 2.2 – Companion | |  |  |  |  |  |
|  | Provider 1 | 2.87 | 4.20 | 3.40 | 3.87 | 3.58 |
|  | Provider 2 | 4.00 | 1.00 | 4.00 | 4.60 | 3.40 |
|  | Provider 3 | 4.20 | 4.33 | 4.60 | 3.80 | 4.23 |
| 2.3 – Disrupted | |  |  |  |  |  |
|  | Provider 1 | 3.87 | —¹ | 4.00 | 4.47 | 4.11 |
|  | Provider 2 | 3.60 | —¹ | 2.20 | 3.87 | 3.22 |
|  | Provider 3 | 4.20 | —¹ | 4.67 | 3.93 | 4.27 |
| 2.4 – Evasive | |  |  |  |  |  |
|  | Provider 1 | 2.73 | 4.47 | 3.93 | 4.40 | 3.88 |
|  | Provider 2 | 3.33 | 4.20 | 4.53 | 3.00 | 3.77 |
|  | Provider 3 | 4.20 | 4.33 | 4.80 | 4.07 | 4.35 |
| 2.5 – Dialect (Gronings) | |  |  |  |  |  |
|  | Provider 1 | 3.07 | 1.00 | 1.00 | 4.13 | 2.30 |
|  | Provider 2 | 3.07 | 4.53 | 3.73 | 3.20 | 3.63 |
|  | Provider 3 | 2.73 | 2.20 | 4.33 | 3.20 | 3.12 |

**Multimedia Appendix 2. Section-level documentation quality scores by script scenario and provider in surgical oncology.**

Values represent mean rater-averaged scores on a 1–5 scale, with higher scores indicating better performance. For each provider and script scenario, note-section scores summarize rater-averaged section–domain evaluations across completeness, correctness, and conciseness. The overall mean across note sections represents the mean across the evaluated note sections. A score of 1 indicates that no usable output was generated for the corresponding note section and was therefore assigned the lowest possible value.
¹ In Script 2.3, the physical examination was intentionally omitted from the script; accordingly, no provider output was expected or scored for this section, and the overall mean was calculated across the evaluated note sections only.
