# Supplementary material for: Three Ambient Listening Systems in Simulated Dutch-Language Multispeaker and Multidialect Clinical Encounters: Comparative Study
Source: JMIR Med Inform. 2026 Oct 16;14:e94586. doi: 10.2196/94586 (PMC13722924; doi:10.2196/94586)
Supplement: Supplementary file 3 [file medinform_v14i1e94586_app3.docx]

**Multimedia Appendix 3. Baseline scripts for otolaryngology and surgical oncology (English translation).**

**Script 1.1**

Participants:

- D: ENT physician (Dr Janssen)
- P: Patient (Mr De Vries, 52 years old)

Context:
Mr De Vries visits the ENT physician because of persistent dizziness and ear complaints.

Script:
D: Good morning, Mr De Vries. I’m Dr Janssen, the ENT physician. What brings you in today?
P: Good morning, doctor. I’ve been having dizziness for a while, and my ears also don’t feel quite right.
D: Could you tell me a bit more about the dizziness? When does it occur, and what exactly does it feel like?
P: It comes and goes. Sometimes when I stand up, but also sometimes when I’m just sitting still. The worst part is that it feels as if the room is spinning around me. Very unpleasant and sometimes even frightening.
D: I understand. How long have you been experiencing this?
P: It’s been going on for about six weeks now. At first I thought it would go away on its own, but over the last two weeks it has only gotten worse.
D: That does sound unpleasant. You also said that your ears don’t feel quite right. Could you explain that a bit more?
P: Yes, my right ear feels a bit full, as if there is water in it. And I can also hear less well on that side.
D: Do you also have tinnitus or a constant sound in your ear, such as ringing or buzzing?
P: No, not that.
D: And do you also have ear pain or fluid coming from the ear?
P: No, I don’t have ear pain or discharge.
D: Do you also have headache or neck pain along with the dizziness?
P: No headache. I do have some stiffness in my neck, but I don’t know whether that has anything to do with it.
D: Are there moments when the dizziness gets worse?
P: Yes, if I move my head quickly, it is at its worst. And when I lie flat, I sometimes get really nauseous. At night I try to sleep on my side, which helps a bit.
D: Understandable that you do that. Have you recently had a cold or an ear infection?
P: No, not that I know of. This really seems to have come out of nowhere.
D: Have you undergone any ENT procedures in the past?
P: Yes, when I was a child my tonsils were removed.
D: Are you taking any medications?
P: No, just a multivitamin that I take daily.
D: Do you have any intoxications such as smoking, alcohol, or drugs?
P: I used to smoke, but not anymore. I drink one glass of wine every day, but I don’t use drugs.
D: Okay. I would like to do a few examinations. In any case, I want to look in your ears and do a balance test. Is that okay?
P: Yes, that’s fine. I’m glad it’s being looked at.
D: Good. I’m first going to look in your ears with the otoscope. Could you tilt your head slightly?

*(Short pause while the physician performs the examination, physician murmurs something)*

D: On the left I see an intact eardrum and an aerated middle ear. On the right I also see an intact eardrum, but I do see some fluid behind the right eardrum. The middle ear is otherwise aerated. This may explain the full feeling and the reduced hearing on that side.
D: I am now going to perform the Dix-Hallpike manoeuvre to investigate your dizziness. What I’m going to do now is quickly tilt your head backward while you are lying down. I will turn your head to the right. You may feel some dizziness during this, but it usually lasts only briefly. Let me know if you experience anything.

*(Dix-Hallpike manoeuvre is performed, physician murmurs again)*

P: Yes, I feel dizzy now when my head is tilted to the right, just like I experienced before.
D: That is clear, this reaction is consistent with what I expected. I also saw your eyes deviate. You may remain lying down quietly until the dizziness subsides. Once you feel better, you may sit up again.

*(Patient sits up)*

D: Based on what I have seen, I think you have BPPV, specifically in the right posterior canal. This stands for Benign Paroxysmal Positional Vertigo. It is caused by small crystals in your balance organ that have become dislodged and ended up in the wrong place. This causes short, severe attacks of dizziness with certain head movements.
P: Oh, that does sound like what I’m experiencing. Can it be treated?
D: Yes, fortunately there is an effective treatment. We can treat this with specific manoeuvres, such as the Epley manoeuvre. I will arrange an appointment for you with the doctor’s assistant to perform this manoeuvre; you should be able to go there later this week.
P: That sounds good. How long does it usually take before something like this goes away?
D: With the Epley manoeuvre, you will usually notice improvement in the dizziness within a few weeks. Are there things in your daily life that are difficult now because of these complaints?
P: Well, I don’t really dare to drive anymore. And climbing stairs is difficult too. I feel quite limited because of it.
D: That is very understandable. I would advise you to be cautious with driving and strenuous activities, such as climbing stairs, for the time being. Once the treatment starts to work, you can gradually build this up again.
P: Okay, thank you. Are there any other things I should pay attention to?
D: Pay attention to whether your symptoms get worse or if new symptoms appear. Avoid sudden head movements, because that can worsen the dizziness. If you become dizzy, sit or lie down quietly until it passes.
P: Understood. I’m glad we now know what it is and that something can be done about it.
D: Indeed, there are good treatment options. Do you have any other questions for now?
P: No, I think everything is clear. When will I see you again?
D: I would like to see you again in six weeks for a follow-up. We will then see how the treatment has gone and whether any adjustments are needed. If the symptoms worsen or if you are concerned, you can contact us sooner.
P: I will do that. Thank you, doctor, see you in six weeks.
D: You’re welcome, Mr De Vries. Take care and goodbye.

**Script 2.1**

Participants:

- D: Surgeon (Dr de Boer, oncological surgeon)
- P: Patient (Mr van der Meer, 62 years old)

Context:
Mr van der Meer visits the oncological surgeon for a follow-up consultation after a recent colonoscopy and biopsy showed colorectal carcinoma.

Script:
D: Good afternoon, Mr van der Meer. I’m Dr de Boer, the oncological surgeon. How are you today?
P: Good afternoon, doctor. To be honest, I’m quite nervous.
D: That is very understandable. Could you first tell me how you have been feeling lately?
P: Well, I feel quite tired. And I’m still having that abdominal pain and changes in my stool. Oh, and I’ve also lost some weight, about 5 kilos over the past two months, I think.
D: The colonoscopy and biopsy showed that you have bowel cancer.

*The physician pauses; the patient reacts emotionally.*

D: We see that you have what we call a moderately differentiated adenocarcinoma in the sigmoid, which is the last part of the large intestine. The tumor is about 3 cm in size and appears to have infiltrated the bowel wall.
P: Moderately differentiated? What exactly does that mean?
D: Differentiation indicates how much the cancer cells resemble normal bowel cells. Moderately differentiated is in between well differentiated and poorly differentiated. This affects how aggressive the tumor may be.
P: Can it be cured? What are the next steps now?
D: We need a CT scan of your abdomen and chest to see whether the cancer has spread. We also want to perform an MRI of the pelvis to assess the local extent more precisely. This helps us determine the stage of the cancer and plan the treatment and surgery.
P: Okay. So surgery is necessary?
D: Yes, in your case we want to perform a sigmoid resection, in which we remove the affected part of the bowel and reconnect the healthy ends.
P: That sounds like a major operation. Are there risks involved?
D: Every operation has risks, but this procedure is performed regularly. The main risks are bleeding, infection, and in rare cases a so-called anastomotic leak. There is also a chance that a stoma may be needed; usually this is temporary.
P: A stoma? What does that involve?
D: A stoma is an artificial opening for stool on the abdomen. It is usually temporary and is created to allow the bowel to heal. In most cases this is not necessary, but we do need to inform you about it.
P: And what about chemotherapy?
D: Whether chemotherapy is needed depends on the stage of the cancer. If the CT scan shows metastases, or if the operation shows that the lymph nodes are involved, then additional chemotherapy is usually indicated.
P: How quickly does all this need to happen?
D: We aim to operate within two weeks after the diagnosis. So we will schedule the additional scans as soon as possible, preferably later this week. After that, we will discuss the results in our multidisciplinary team and make a definitive treatment plan.
P: That’s all happening very fast. I don’t know whether I’m ready for it.
D: I understand that this is overwhelming. A lot is happening at once. We have an oncology nurse who can guide you and answer all your questions; you will speak with her shortly and again later this week.
P: Are there things I can already do now to prepare?
D: Certainly. It is important to stop smoking if you smoke, to eat healthily, and to exercise moderately within your limits. This can speed recovery after surgery. We will also soon do preoperative screening to make sure you are fit enough for the procedure.
P: I don’t smoke, but I will try to be a bit more active. How long does recovery after the operation usually take?
D: After a sigmoid resection without complications, patients can usually go home after 5 to 7 days. Full recovery takes about 6 to 8 weeks. But this differs from person to person and depends on factors such as age and general health.
P: Thank you. It’s a lot of information to process. I’m afraid I’ve already forgotten half of it.
D: That is completely normal, Mr van der Meer. You will also receive all the information in writing, and our oncology nurse will go through everything with you again calmly. I was also wondering whether you have had cancer before, or whether cancer runs in your family?
P: Not as far as I know.
D: And medication use, specifically blood thinners?
P: I don’t use blood thinners, but I do take a pill for cholesterol, I think it’s called simvastatin.

D: Lastly, I just want to briefly listen to your abdomen. You can stay seated; you only need to lift your shirt a little.

*The physician listens with a stethoscope.*

D: You can lower your shirt again. I hear normal peristalsis, which is good. The oncology nurse will come in shortly to arrange the practical matters and answer any questions that may come up later. We will see each other again after the scans to discuss the definitive treatment plan. Is that okay?
P: Yes, that seems fine. Thank you for your explanation and patience.
D: You’re welcome, Mr van der Meer. We will do everything we can together to give you the best treatment. I wish you strength in the coming period.
P: Thank you, doctor. Goodbye.
