# Supplementary material for: Three Ambient Listening Systems in Simulated Dutch-Language Multispeaker and Multidialect Clinical Encounters: Comparative Study
Source: JMIR Med Inform. 2026 Oct 16;14:e94586. doi: 10.2196/94586 (PMC13722924; doi:10.2196/94586)
Supplement: Supplementary file 4 [file medinform_v14i1e94586_app4.docx]

**Multimedia Appendix 4. Interrater agreement for clinical documentation quality scores using Gwet agreement coefficient 2 (Gwet AC-2).**

| Section | Level | n | AC2 | 95% CI |
| --- | --- | --- | --- | --- |
| **Overall (8 raters)** | | 351 | 0.827 | 0.806–0.847 |
| **Juniors only (4 raters)** | | 351 | 0.828 | 0.807–0.851 |
| **Juniors + single senior** | |  |  |  |
|  | Senior A | 105 | 0.844 | 0.795–0.886 |
|  | Senior B | 69 | 0.795 | 0.749–0.841 |
|  | Senior C | 105 | 0.826 | 0.788–0.863 |
|  | Senior D | 72 | 0.847 | 0.718–0.880 |
| **By domain (all raters)** | |  |  |  |
|  | Completeness | 117 | 0.780 | 0.743–0.811 |
|  | Conciseness | 117 | 0.849 | 0.809–0.881 |
|  | Correctness | 117 | 0.860 | 0.822–0.895 |
| **By note section (all raters)** | |  |  |  |
|  | Patient history | 90 | 0.781 | 0.730–0.830 |
|  | Physical examination | 81 | 0.835 | 0.796–0.870 |
|  | Diagnostic impression | 90 | 0.882 | 0.847–0.917 |
|  | Treatment plan | 90 | 0.824 | 0.774–0.865 |

Agreement is reported overall across all 8 raters, among the 4 junior raters, for combinations of the 4 junior raters with a single senior rater, and stratified by scoring domain and note section. Ninety-five percent confidence intervals (95% CI) are shown. Scores were based on a 1–5 ordinal rating scale.
